# Novel Mouse Xenograft Models Reveal a Critical Role of CD4^+^ T Cells in the Proliferation of EBV-Infected T and NK Cells

**DOI:** 10.1371/journal.ppat.1002326

**Published:** 2011-10-20

**Authors:** Ken-Ichi Imadome, Misako Yajima, Ayako Arai, Atsuko Nakazawa, Fuyuko Kawano, Sayumi Ichikawa, Norio Shimizu, Naoki Yamamoto, Tomohiro Morio, Shouichi Ohga, Hiroyuki Nakamura, Mamoru Ito, Osamu Miura, Jun Komano, Shigeyoshi Fujiwara

**Affiliations:** 1 Department of Infectious Diseases, National Research Institute for Child Health and Development, Tokyo, Japan; 2 Department of Hematology, Tokyo Medical and Dental University, Tokyo, Japan; 3 Department of Pathology, National Center for Child Health and Development, Tokyo, Japan; 4 Department of Virology, Division of Medical Science, Medical Research Institute, Tokyo Medical and Dental University, Tokyo, Japan; 5 AIDS Research Center, National Institute of Infectious Diseases, Tokyo, Japan; 6 Department of Pediatrics and Developmental Biology, Tokyo Medical and Dental University, Tokyo, Japan; 7 Department of Perinatal and Pediatric Medicine, Graduate School of Medical Sciences, Kyushu University, Fukuoka, Japan; 8 Central Institute for Experimental Animals, Kawasaki, Japan; University of Texas Health Science Center San Antonio, United States of America

## Abstract

Epstein-Barr virus (EBV), a ubiquitous B-lymphotropic herpesvirus, ectopically infects T or NK cells to cause severe diseases of unknown pathogenesis, including chronic active EBV infection (CAEBV) and EBV-associated hemophagocytic lymphohistiocytosis (EBV-HLH). We developed xenograft models of CAEBV and EBV-HLH by transplanting patients' PBMC to immunodeficient mice of the NOD/Shi-*scid*/IL-2Rγ^null^ strain. In these models, EBV-infected T, NK, or B cells proliferated systemically and reproduced histological characteristics of the two diseases. Analysis of the TCR repertoire expression revealed that identical predominant EBV-infected T-cell clones proliferated in patients and corresponding mice transplanted with their PBMC. Expression of the EBV nuclear antigen 1 (EBNA1), the latent membrane protein 1 (LMP1), and LMP2, but not EBNA2, in the engrafted cells is consistent with the latency II program of EBV gene expression known in CAEBV. High levels of human cytokines, including IL-8, IFN-γ, and RANTES, were detected in the peripheral blood of the model mice, mirroring hypercytokinemia characteristic to both CAEBV and EBV-HLH. Transplantation of individual immunophenotypic subsets isolated from patients' PBMC as well as that of various combinations of these subsets revealed a critical role of CD4^+^ T cells in the engraftment of EBV-infected T and NK cells. In accordance with this finding, in vivo depletion of CD4^+^ T cells by the administration of the OKT4 antibody following transplantation of PBMC prevented the engraftment of EBV-infected T and NK cells. This is the first report of animal models of CAEBV and EBV-HLH that are expected to be useful tools in the development of novel therapeutic strategies for the treatment of the diseases.

## Author Summary

Epstein-Barr virus (EBV) is a ubiquitous human herpesvirus that infects more than 90% of the adult human population in the world. EBV usually infects B lymphocytes and does not produce symptoms in infected individuals, but in rare occasions it infects T or NK lymphocytes and causes severe diseases such as chronic active EBV infection (CAEBV) and EBV-associated hemophagocytic lymphohistiocytosis (EBV-HLH). We developed mouse models of these two human diseases in which EBV-infected T or NK lymphocytes proliferate in mouse tissues and reproduce human pathologic conditions such as overproduction of small proteins called “cytokines” that produce inflammatory responses in the body. These mouse models are thought to be very useful for the elucidation of the pathogenesis of CAEBV and EBV-HLH as well as for the development of therapeutic strategies for the treatment of these diseases. Experiments with the models demonstrated that a subset of lymphocytes called CD4-positive lymphocytes are essential for the proliferation of EBV-infected T and NK cells. This result implies that removal of CD4-positive lymphocytes or suppression of their functions may be an effective strategy for the treatment of CAEBV and EBV-HLH.

## Introduction

Epstein-Barr virus (EBV) is a ubiquitous γ-herpesvirus that infects more than 90% of the adult population in the world. EBV is occasionally involved in the pathogenesis of malignant tumors, such as Burkitt lymphoma, Hodgkin lymphoma, and nasopharyngeal carcinoma, along with the post-transplantation lymphoproliferative disorders in immunocompromised hosts. Although EBV infection is asymptomatic in most immunologically competent hosts, it sometimes causes infectious mononucleosis (IM), when primarily infecting adolescents and young adults [1]. EBV infects human B cells efficiently in vitro and transform them into lymphoblastoid cell lines (LCLs) [2]. Experimental infection of T and NK cells, in contrast, is practically impossible except in limited conditions [3], [4]. Nevertheless, EBV has been consistently demonstrated in T or NK cells proliferating monoclonally or oligoclonally in a group of diseases including chronic active EBV infection (CAEBV) and EBV-associated hemophagocytic lymphohistiocytosis (EBV-HLH) [5], [6], [7], [8], [9], [10]. CAEBV, largely overlapping the systemic EBV^+^ T-cell lymphoproliferative diseases of childhood defined in the WHO classification of lymphomas [11], is characterized by prolonged or relapsing IM-like symptoms, unusual patterns of antibody responses to EBV, and elevated EBV DNA load in the peripheral blood [12], [13], [14]. CAEBV has a chronic time course with generally poor prognosis; without a proper treatment by hematopoietic stem cell transplantation, the majority of cases eventually develop malignant lymphoma of T or NK lineages, multi-organ failure, or other life-threatening conditions. Monoclonal or oligoclonal proliferation of EBV-infected T and NK cells, an essential feature of CAEBV, implies its malignant nature, but other characteristics of CAEBV do not necessarily support this notion. For example, EBV-infected T or NK cells freshly isolated from CAEBV patients, as well as established cell lines derived from them, do not have morphological atypia and do not engraft either in nude mice or *scid* mice (Shimizu, N., unpublished results). Clinically, CAEBV has a chronic time course and patients may live for many years without progression of the disease [15]. Although patients with CAEBV do not show signs of explicit immunodeficiency, some of them present a deficiency in NK-cell activity or in EBV-specific T-cell responses, implying a role for subtle immunodeficiency in its pathogenesis [16], [17], [18].

EBV-HLH is the most common and the severest type of virus-associated HLH and, similar to CAEBV, characterized by monoclonal or oligoclonal proliferation of EBV-infected T (most often CD8^+^ T) cells [5], [6]. Clinical features of EBV-HLH include high fever, pancytopenia, coagulation abnormalities, hepatosplenomegaly, liver dysfunction, and hemophagocytosis [19]. Overproduction of cytokines by EBV-infected T cells as well as by activated macrophages and T cells reacting to EBV is thought to play a central role in the pathogenesis [20]. Although EBV-HLH is an aggressive disease requiring intensive clinical interventions, it may be cured, in contrast to CAEBV, by proper treatment with immunomodulating drugs [21]. No appropriate animal models have been so far developed for either CAEBV or EBV-HLH.

NOD/Shi-*scid/*IL-2Rγ^null^ (referred here as NOG) is a highly immunodeficient mouse strain totally lacking T, B, and NK lymphocytes, and transplantation of human hematopoietic stem cells to NOG mice results in reconstitution of human immune system components, including T, B, NK cells, dendritic cells, and macrophages [22], [23]. These so called humanized mice have been utilized as animal models for the infection of certain human viruses targeting the hemato-immune system, including human immunodeficiency virus 1 (HIV-1) and EBV [24], [25], [26], [27], [28], [29], [30]. Xenotransplantation of human tumor cells to NOG mice also provided model systems for several hematologic malignancies [31], [32], [33]. To facilitate investigations on the pathogenesis of CAEBV and EBV-HLH and assist the development of novel therapeutic strategies, we generated mouse models of these two EBV-associated diseases by transplanting NOG mice with PBMC isolated from patients with the diseases. In these models, EBV-infected T, NK, or B cells engrafted in NOG mice and reproduced lymphoproliferative disorder similar to either CAEBV or EBV-HLH. Further experiments with the models revealed a critical role of CD4^+^ T cells in the in vivo proliferation of EBV-infected T and NK cells.

## Results

### Engraftment of EBV-infected T and NK cells in NOG mice following xenotransplantation with PBMC of CAEBV patients

**Figure 1:**
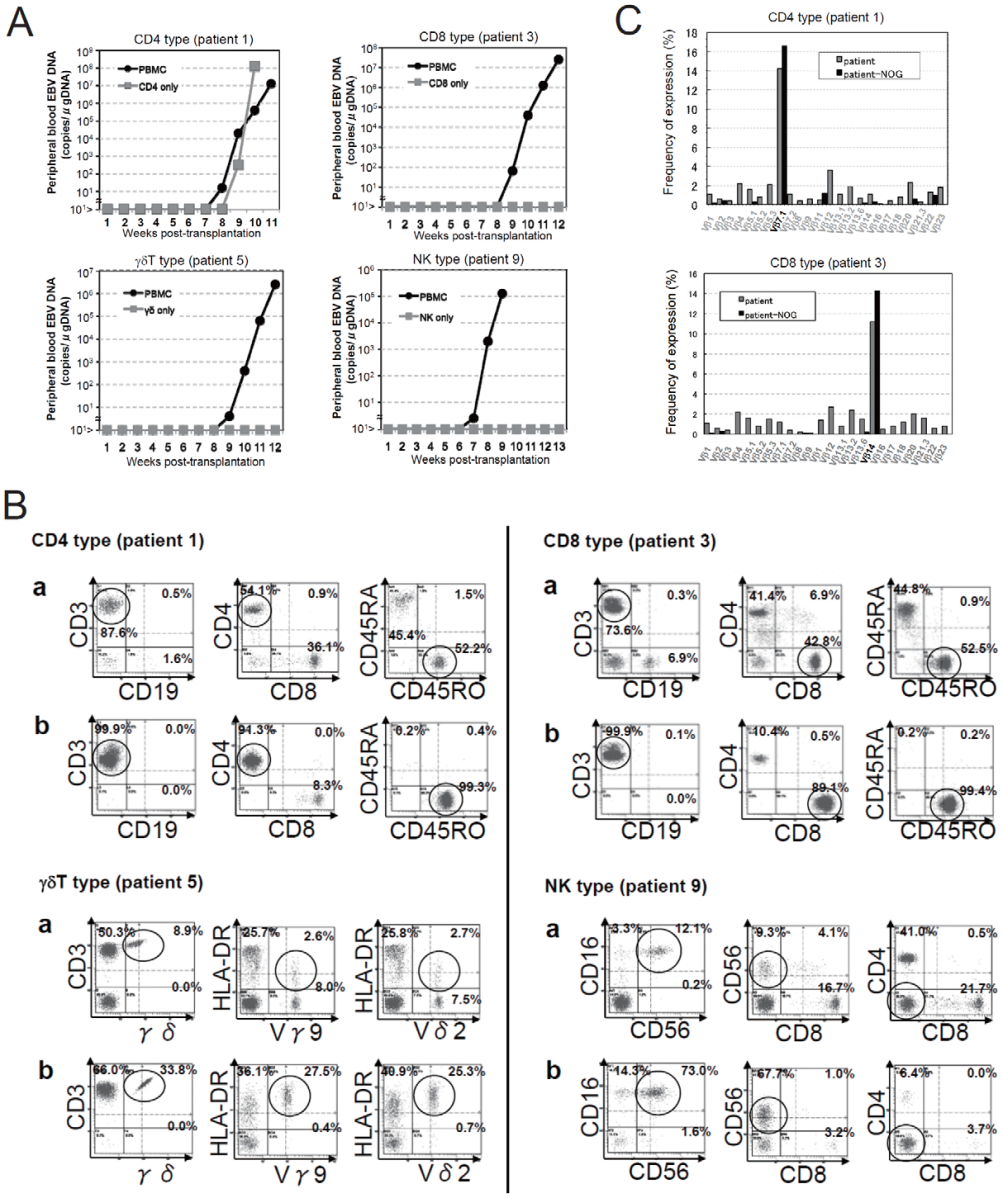
Engraftment of EBV-infected T or NK cells in NOG mice following transplantation with PBMC of patients with CAEBV. A. Measurement of EBV DNA levels. PBMC obtained from the CAEBV patients 1 (CD4 type), 3 (CD8 type), 5 (γδT type), and 9 (NK type) were injected intravenously to NOG mice and EBV DNA load in their peripheral blood was measured weekly by real-time PCR. The results of transplantation with whole PBMC or with isolated EBV DNA-containing cell fraction are shown. B. Flow-cytometric analysis on the expression of surface markers in the peripheral blood lymphocytes of patients (a) with CAEBV and NOG mice (b) that received PBMC from them. Human lymphocytes gated by the pattern of side scatter and human CD45 expression were further analyzed for the expression of various surface markers indicated in the figures. The results from the patients 1, 3, 5, and 9, and the corresponding mice that received their respective PBMC are shown. Circles indicate the fractions that contained EBV DNA. C. Analysis on the expression of TCR Vβ repertoire. Peripheral blood lymphocytes obtained from the patients 1 (CD4 type) and 3 (CD8 type), and from the corresponding mice that received their respective PBMC were analyzed for the expression of Vβ alleles. The percentages of T cells expressing each Vβ allele are shown for the patients (grey bars) and the mice (black bars).

Depending on the immunophenotypic subset in which EBV causes lymphoproliferation, CAEBV is classified into the T-cell and NK-cell types, with the former being further divided into the CD4, CD8, and γδT types. The nine patients with CAEBV examined in this study are characterized in Table 1 and include all these four types. Intravenous injection of 1−4×10^6^ PBMC isolated from these nine patients resulted in successful engraftment of EBV-infected T or NK cells in NOG mice in a reproducible manner (Table 1). The results with the patient 1 (CD4 type), patient 3 (CD8 type), patient 5 (γδT type), and patient 9 (NK type) are shown in Figure 1. Seven to nine weeks post-transplantation, EBV DNA was detected in the peripheral blood of recipient mice and reached the levels of 10^5^–10^8^ copies/µg DNA (Figure 1A). By contrast, no engraftment of EBV-infected cells was observed when immunophenotypic fractions containing EBV DNA were isolated from PBMC and injected to NOG mice (Figure 1A and Table 2). An exception was the CD4^+^ T-cell fraction isolated from patients with the CD4 type CAEBV, that reproducibly engrafted when transplanted without other components of PBMC (Figure 1A, Table 2). Flow cytometry revealed that the major population of engrafted cells was either CD4^+^, CD8^+^, TCRγδor CD16^+^CD56^+^, depending on the type of the donor CAEBV patient (Figure 1B). EBV-infected cells of identical immunophenotypes were found in the patients and the corresponding mice that received their respective PBMC (Figure 1B). Although human cells of multiple immunophenotypes were present in most recipient mice, fractionation by magnetic beads-conjugated antibodies and subsequent real-time PCR analysis detected EBV DNA only in the predominant immunophenotypes that contained EBV DNA in the original patients (Figure 1B, Table 1). The EBV DNA load observed in individual lymphocyte subsets in the patient 3 and a mouse that received her PBMC is shown as supporting data (Table S1). When PBMC from three healthy EBV-carriers were injected intravenously to NOG mice, as controls, no EBV DNA was detected from either the peripheral blood, spleen, or liver (data not shown). Histological analyses of the spleen and the liver of these control mice identified no EBV-encoded small RNA (EBER)-positive cells, although some CD3-positive human T cells were observed (Figure S2). Analysis of TCR Vβ repertoire demonstrated an identical predominant T-cell clone in patients (patients 1 and 3) and the corresponding mice that received their PBMC (Figure 1C). The general condition of most recipient mice deteriorated gradually in the observation period of eight to twelve weeks, with loss of body weight (Figure S1), ruffled hair, and inactivity.

**Table 1:** Patients with EBV-T/NK LPD and the results of xenotransplantation of their PBMC to NOG mice.

Patient number	Diagnosis	Sex	Age	Type of infected cells	1EBV DNA load in the patients	2Engrafted cells in mice	3Engraftment	1EBV DNA load in mice
1	CAEBV	F	25	CD4	9.2×10^5^	CD4, CD8	3/3	1.0∼3.8×10^7^
2	CAEBV	M	46	CD4	1.3∼7.2×10^5^	CD4, CD8	2/2, 3/3	2.6∼10×10^5^
3	CAEBV	F	35	CD8	2.1∼78×10^5^	CD8, CD4	2/2, 2/2	1.1∼33×10^6^
4	CAEBV	M	28	CD8	8.2×10^5^	CD8, CD4	3/3	1.1∼2.5×10^6^
5	CAEBV	M	10	γδT	2.2×10^6^	γδT, CD4, CD8	2/2	3.8∼6.5×10^6^
6	CAEBV	F	15	γδT	6.2×10^5^	γδT, CD4, CD8	2/2	2.2∼11×10^5^
7	CAEBV	M	13	NK	1.1∼6.7×10^5^	NK, CD4, CD8	2/2, 2/2	0.6∼15×10^4^
8	CAEBV	F	13	NK	6.3×10^6^	NK, CD4, CD8	3/3, 2/2	0.8∼1.9×10^5^
9	CAEBV	M	8	NK	1.2∼8.7×10^5^	NK, CD4, CD8	2/2, 3/3	1.8∼7.2×10^5^
10	EBV-HLH	M	10	CD8	2.8∼38×10^4^	CD8, CD4	2/2, 2/2	6.5∼9.9×10^4^
11	EBV-HLH	M	50	CD8	6.2×10^5^	CD8, CD4	4/4	7.0∼45×10^4^
12	EBV-HLH	M	1	CD8	3.1×10^5^	CD8, CD4	2/2	6.0∼9.1×10^4^
13	EBV-HLH	M	64	CD8	3.2∼3.9×10^5^	CD8, CD4	2/2, 2/2	5.0∼30×10^5^

1 EBV DNA copies/µg DNA in the peripheral blood.

2 EBV DNA was detected only in the cells of the underlined subsets.

3 Number of mice with successful engraftment per number of recipient mice is shown for each experiment.

**Table 2:** Results of xenotransplantation with subsets of PBMC obtained from CAEBV patients.

Number of patient	Diagnosis	Phenotype of infected cells	Cell fraction transplanted	Number of transplanted cells	Engraftment
1	CAEBV	CD4	PBMC	2×10^6^	+
			CD4	2×10^6^	+
			PBMC-CD4	3×10^6^	−
			PBMC-CD8	2×10^6^	+
			PBMC-CD56	2×10^6^	+
			PBMC-CD14	2×10^6^	+
			PBMC-CD19	2×10^6^	+
3	CAEBV	CD8	PBMC	2×10^6^	+
			CD8	3×10^6^	−
			PBMC-CD4	3×10^6^	−
			PBMC-CD8	3×10^6^	−
			PBMC-CD56	2×10^6^	+
			PBMC-CD14	2×10^6^	+
			PBMC-CD19	2×10^6^	+
5	CAEBV	γδT	PBMC	2×10^6^	+
			γδT	3×10^6^	−
			PBMC-CD4	3×10^6^	−
			PBMC-γδT	3×10^6^	−
			PBMC- CD8	3×10^6^	+
			PBMC-CD56	3×10^6^	+
			PBMC-CD14	3×10^6^	+
			PBMC-CD19	3×10^6^	+
9	CAEBV	NK	PBMC	2×10^6^	+
			NK	3×10^6^	−
			PBMC-CD4	3×10^6^	−
			PBMC-CD8	3×10^6^	+
			PBMC-CD56	3×10^6^	−
			PBMC-CD14	3×10^6^	+
			PBMC-CD19	3×10^6^	+
11	EBV-HLH	CD8	PBMC	2×10^6^	+
			PBMC-CD4	4×10^6^	−

**Figure 2:**
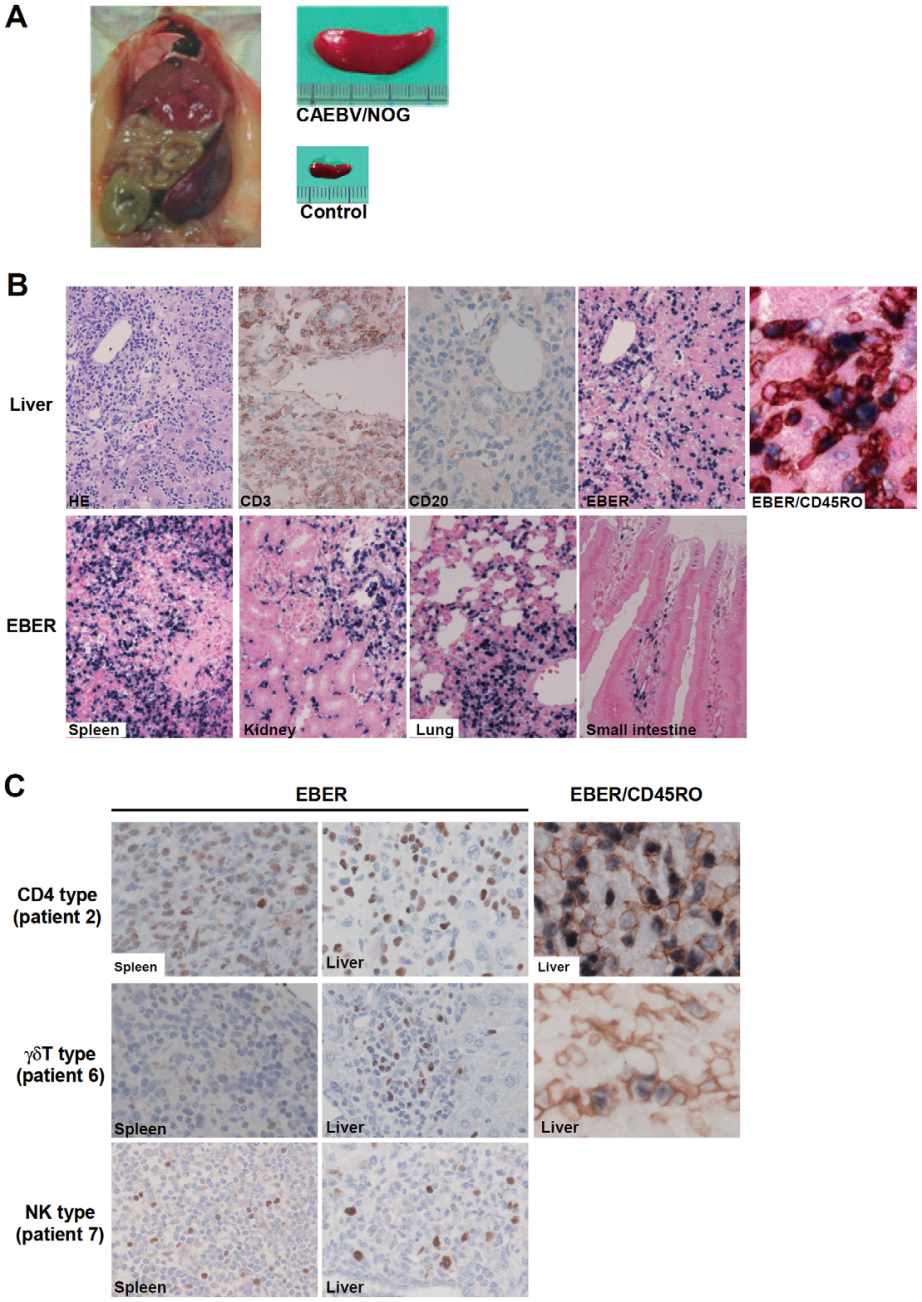
Pathological and immunochemical analyses on NOG mice transplanted with PBMC from CAEBV patients. A. Photographs of a model mouse showing splenomegaly and of the excised spleen. This mouse was transplanted with PBMC from the CAEBV patient 3 (CD8 type). Spleen from a control NOG mouse is also shown. B. Photomicrographs of various tissues of a mouse that received PBMC from the patient 3 (CD8 type). Upper panels: liver tissue was stained with hematoxylin-eosin (HE), antibodies specific to human CD3 or CD20, or by ISH with an EBER probe; the rightmost panel is a double staining with EBER and human CD45RO. Bottom panels: EBER ISH in the spleen, kidney, lung, and small intestine. Original magnification is ×200, except for EBER/CD45RO, that is ×400. C. Photomicrographs of the spleen and liver tissues obtained from NOG mice transplanted with PBMC from the CAEBV patients 2 (CD4 type), 6 (γδT type) or 7 (NK type). Tissues were stained by EBER-ISH or by double staining with EBER-ISH and human CD45RO. Original magnification ×600.

NOG mice engrafted with EBV-infected T or NK cells were sacrificed for pathological and virological analyses between eight and twelve weeks post-transplantation. On autopsy, the majority of mice presented with splenomegaly, with slight hepatomegaly in occasional cases (Figure 2A). Histopathological findings obtained from a representative mouse (recipient of PBMC from the patient 3 (CD8 type)) are shown in Figure 2B and reveal infiltration of human CD3^+^CD20^−^ cells to major organs, including the spleen, liver, lungs, kidneys, and small intestine. These cells were positive for both EBER and human CD45RO, indicating that they are EBV-infected human T cells (Figure 2B). In contrast, no EBV-infected T cells were found in mice transplanted with PBMC isolated from a normal EBV carrier (Figure S2). Histopathology of a control NOG mouse is shown in Figure S2. Morphologically, EBV-infected cells are relatively small and do not have marked atypia. The infiltration pattern was leukemic and identical with chronic active EBV infection in children [34]. The architecture of the organs was well preserved in spite of marked lymphoid infiltration. The spleen showed marked expansion of periarterial lymphatic sheath owing to lymphocytic infiltration. In the liver, a dense lymphocytic infiltration was observed in the portal area and in the sinusoid. The lung showed a picture of interstitial pneumonitis and the lymphocytes often formed nodular aggregations around bronchioles and arteries. In the kidney, dense lymphocytic infiltration caused interstitial nephritis. In the small intestine, mild lymphoid infiltration was seen in mucosa. Quantification of EBV DNA in the spleen, liver, lymph nodes, lungs, kidneys, adrenals, and small intestine of this mouse revealed EBV DNA at the levels of 1.5–5.1×10^7^ copies/µg DNA. Mice transplanted with PBMC derived from CAEBV of other types exhibited similar infiltration of EBV-infected T or NK cells to the spleen, liver, and other organs (Figure 2C and data not shown).

### EBV-infected T- and NK-cell lines established from CAEBV patients do not engraft in NOG mice

**Figure 3:**
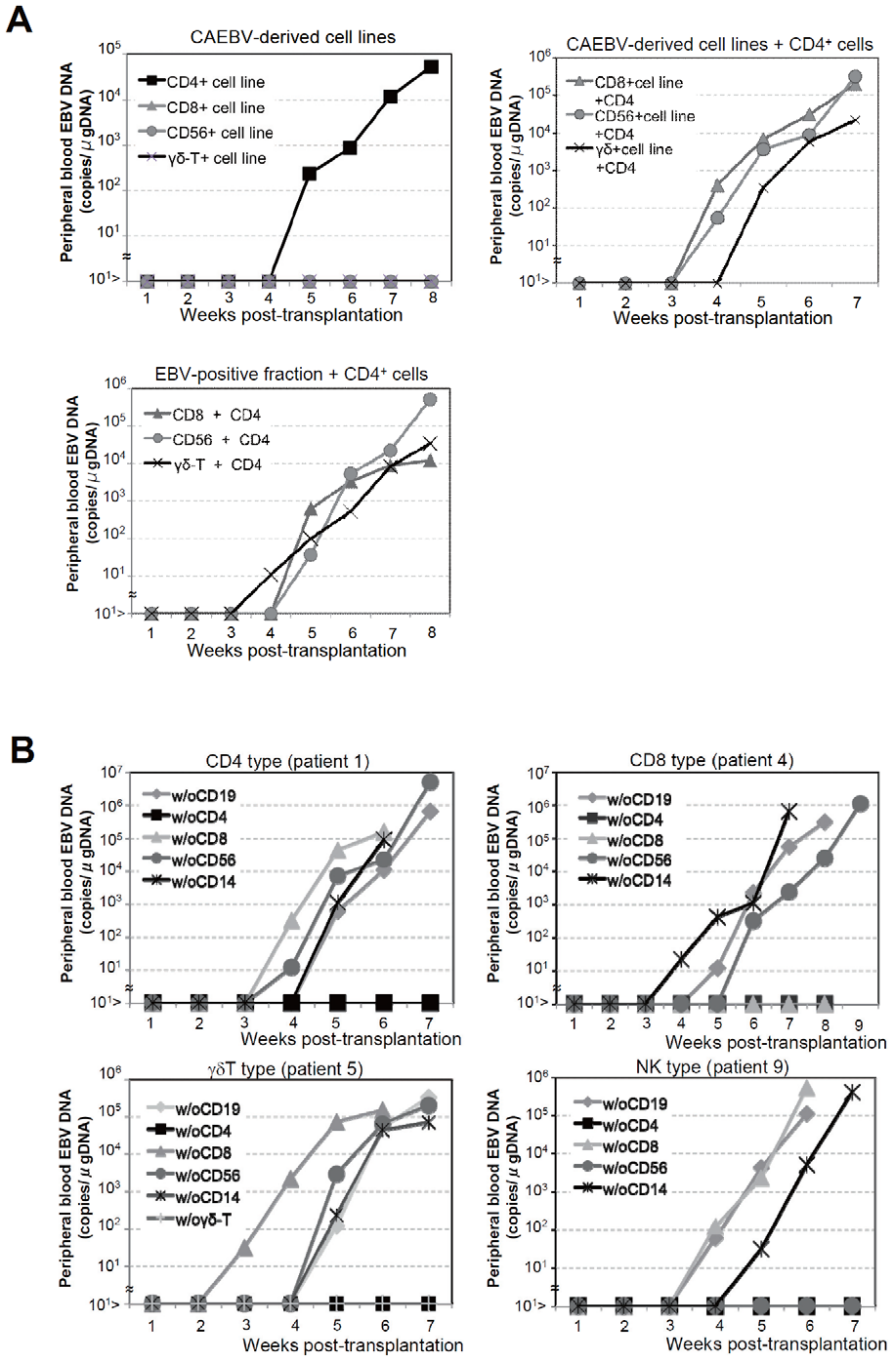
Analysis on the conditions of the engraftment of EBV-infected T and NK cells in NOG mice. A. EBV-infected T or NK cells isolated from patients with CAEBV or cell lines derived from them were injected to NOG mice in the conditions described below. Peripheral blood EBV DNA levels were then measured weekly. Upper-left panel: 5×10^6^ cells of EBV-infected CD4^+^ T, CD8^+^ T, γδT, and CD56^+^ NK cell lines established from the CAEBV patients 1, 4, 6, and 8, respectively, were injected intravenously to NOG mice. Upper-right panel: 5×10^6^ cells of the CD8^+^ T, γδT, and CD56^+^ NK cell lines established from the patients 3, 6, and 8, respectively, were injected intravenously to NOG mice together with autologous CD4^+^ T cells isolated from 5×10^6^ PBMC. Bottom panel: 5×10^6^ cells of the CD8^+^ T, γδT, and CD56^+^ NK fractions isolated freshly from the patients 4, 5, and 7, respectively, were injected intravenously to NOG mice together with autologous CD4^+^ T cells isolated from 5×10^6^ PBMC. B. Transplantation of PBMC devoid of individual immunophenotypic subsets to NOG mice. CD19^+^, CD4^+^, CD8^+^, CD56^+^, or CD14^+^ cells were removed from PBMC obtained from the patient 1 (CD4 type, upper-left panel), 4 (CD8 type, upper-right), 5 (γδT type, bottom-left), and 9 (NK type, bottom-right) and the remaining cells were injected intravenously to NOG mice. Thereafter peripheral blood EBV DNA was determined weekly.

We established EBV-positive cell lines of CD4^+^ T, CD8^+^ T, γδT, and CD56^+^ NK lineages from PBMC of the patients listed in Table 1 by the method described previously [35], and confirmed by flow cytometry that the surface phenotypes of EBV-infected cells in the original patients were retained in these cell lines (data not shown). To test whether these cell lines engraft in NOG mice, 1–4×10^6^ cells were injected intravenously to NOG mice. The results are shown in Figure 3A and indicate that CAEBV-derived cell lines of the CD8^+^ T, γδT, and CD56^+^ NK phenotypes do not engraft in NOG mice. Neither human CD45-positive cells nor EBV DNA were detected in the peripheral blood of the mice up to twelve weeks post-transplantation. When the recipient mice were sacrificed at twelve weeks post-injection, no EBV DNA could be detected in the spleen, liver, bone marrow, mesenteric lymph nodes, and kidneys. In contrast, the CD4^+^ T cell lines derived from the CD4-type patients 1 and 2 engrafted in NOG mice and induced T lymphoproliferation similar to that induced by PBMC isolated freshly from these patients (Figure 3A and data not shown). These results, together with the results of transplantation with EBV-containing subsets of PBMC, indicate that EBV-infected T and NK cells, with the exception of those of the CD4^+^ subset, are not able to engraft in NOG mice, when they are separated from other components of PBMC, suggesting that some components of PBMC are essential for the outgrowth EBV-infected T and NK cells in NOG mice.

### Engraftment of EBV-infected T and NK cells in NOG mice requires CD4^+^ T cells

**Figure 4:**
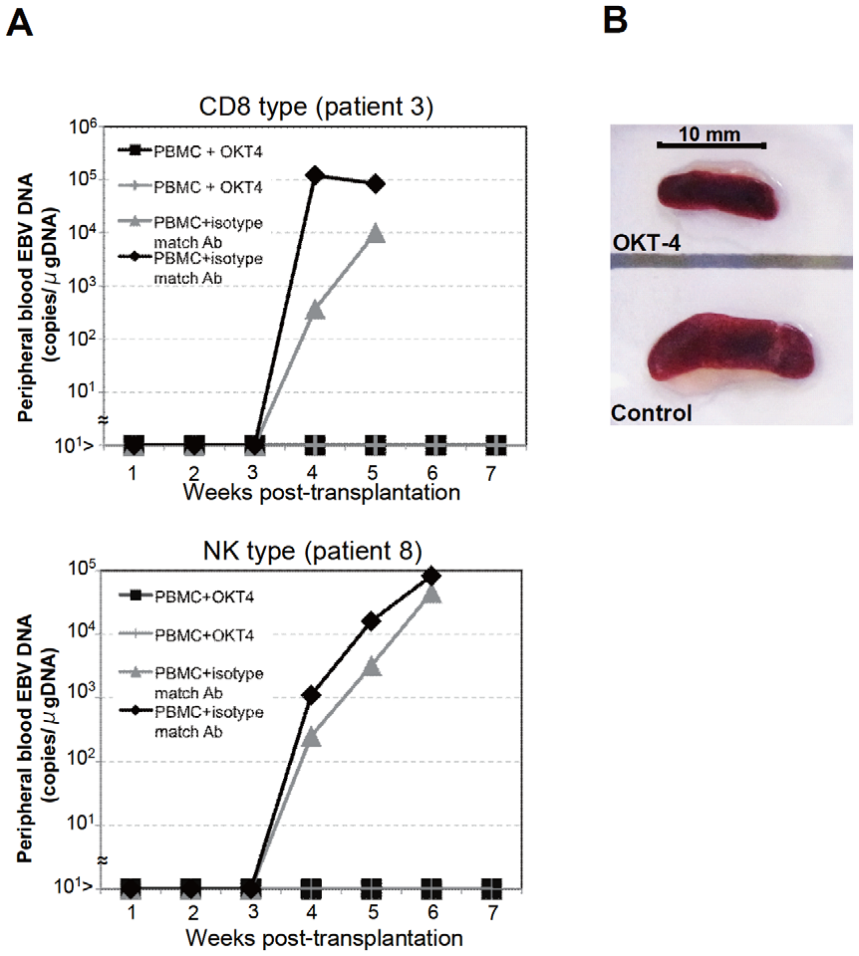
Suppression of the engraftment of EBV-infected T and NK cells by the OKT-4 antibody. PBMC (5×10^6^ cells) isolated from the CAEBV patient 3 (CD8 type) or 8 (NK type) were injected intravenously to NOG mice. The OKT-4 antibody (100 µg/mouse) was administered intravenously on the same day of transplantation and the following three consecutive days. As a control, isotype-matched mouse IgG was injected. A. Changes in the peripheral blood EBV DNA level in the recipient mice. Results with the mice transplanted with PBMC of the patient 3 (top) and of the patient 8 (bottom) are shown. B. Photographs of the spleen of an OKT-4-treated mouse (top) and a control mouse (bottom) taken at autopsy.

To identify the cellular component required for the engraftment of EBV-infected T and NK cells in NOG mice, we transplanted PBMC of CAEBV patients after removing individual immunophenotypic subsets by magnetic beads-conjugated antibodies. The results are shown in Figure 3B and summarized in Table 2. With respect to the patients 3 and 4, in whom CD8^+^ T cells are infected with EBV, removal of CD8^+^ cells from PBMC, as expected, resulted in the failure of engraftment, whereas elimination of CD19^+^, CD56^+^, or CD14^+^ cells did not affect engraftment. Importantly, elimination of CD4^+^ cell fraction, that did not contain EBV DNA, resulted in the failure of engraftment of EBV-infected T cells (Figure 3B and data not shown). In the experiments with the patients 5 and 6, in whom γδT cells were infected, removal CD4^+^ cells that did not contain EBV DNA, as well as that of γδT cells, resulted in the failure of engraftment. Removal of CD8^+^, CD14^+^, CD19^+^, or CD56^+^ cells did not have an influence on the engraftment (Figure 3B and data not shown). Regarding the patients 8 an 9 in whom EBV resided in CD56^+^ NK cells, removal of CD4^+^ as well as CD56^+^ cells resulted in the failure of engraftment, whereas that of CD8^+^, CD19^+^, or CD14^+^ cells did not affect engraftment (Figure 3B and data not shown). In the patients 1 and 2, in whom CD4^+^ T cells were infected, only the removal of CD4^+^ cells blocked the engraftment of EBV-infected cells and depletion of either CD8^+^, CD19^+^, or CD14^+^ cells had no effect (Figure 3B and data not shown). These results suggested that EBV-infected cells of the CD8^+^, γδT, and CD56^+^ lineages require CD4^+^ cells for their engraftment in NOG mice. To confirm this interpretation, we performed complementation experiments, in which EBV-containing fractions of the CD8^+^ (patient 4), γδT (patient 5), or CD56^+^ (patient 7) phenotypes were transplanted together with autologous CD4^+^ cells. The results are shown in Figure 3A and indicate that EBV-infected CD8^+^, γδT, or CD56^+^ cells engraft in NOG mice when transplanted together with CD4^+^ cells. Similarly, when EBV-infected cell lines of the CD8^+^, γδT, and CD16^+^ lineages were injected intravenously to NOG mice together with autologous CD4^+^ cells, these cell lines engrafted to the mice (Figure 3A). Finally, to further confirm the essential role of CD4^+^ cells, we examined the effect of the OKT-4 antibody that depletes CD4^+^ cells in vivo [24]. PBMC isolated from the CAEBV patient 3 (CD8 type) and the patient 8 (NK type) were injected intravenously to NOG mice and OKT-4 was administered intravenously for four consecutive days starting from the day of transplantation. The results are shown in Figure 4 and indicate that OKT-4 can strongly suppress the engraftment of EBV-infected T and NK cells. In the mice treated with OKT-4, no splenomegaly was observed and EBV DNA was not detected either in the peripheral blood, spleen, liver, or lungs at eight weeks post-transplantation.

### Analysis on the EBV gene expression associated with T or NK lymphoproliferation in NOG mice

**Figure 5:**
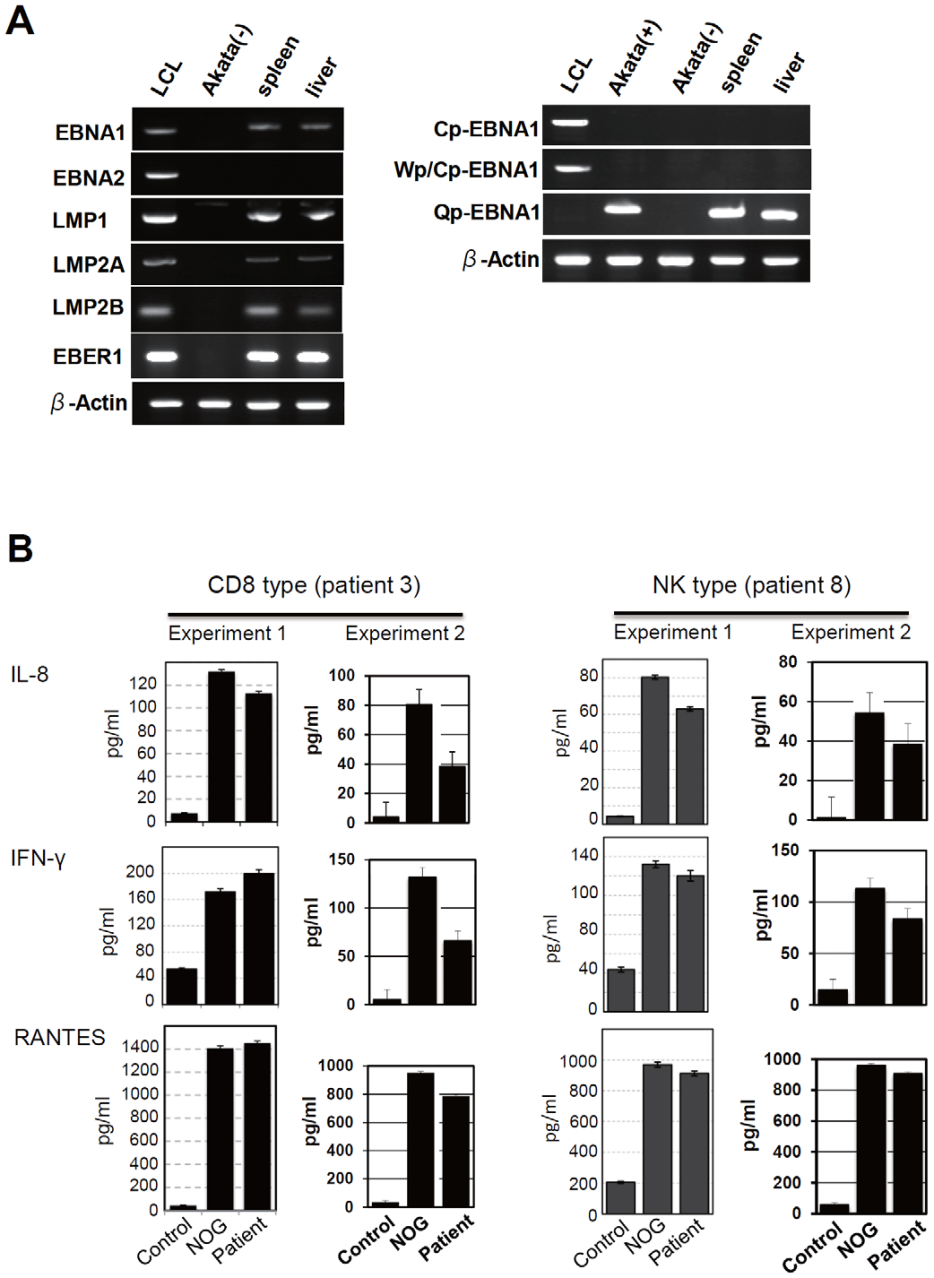
Analyses on the latent EBV gene expression and cytokine production in NOG mice transplanted with PBMC of CAEBV patients. A. EBV gene expression. Total RNA was purified from the spleen and liver of a mouse that received PBMC from the patient 3 (CD8 type) and applied for RT-PCR assay to detect transcripts from the indicated genes. RNA samples from an EBV-transformed B-lymphoblastoid cell line (LCL) and from EBV-negative Akata cell line were used as positive and negative controls, respectively. The primers used in the experiments are shown in Materials and Methods. B. Quantification of plasma levels of human cytokines in patients with CAEBV and corresponding recipient mice. PBMC were isolated from the patients 3 (CD8 type) and 8 (NK type) in two occasions and transplanted to NOG mice. Plasma cytokine levels of the patients were determined when their PBMC were isolated. Plasma cytokine levels of the corresponding recipient mice, prepared on each occasion of PBMC collection, were determined when they were sacrificed. Concentration of human IL-8, IFN-γ, and RANTES were measured by appropriate ELISA kits following the instruction provided by the manufacturer. Plasma samples from healthy adults were used as a control. The bars represent mean values and standard errors from triplicate measurements.

Previous analysis of EBV gene expression in patients with CAEBV revealed the expression of EBNA1, LMP1, and LMP2A with the involvement of the Q promoter in the EBNA genes transcription and no expression of EBNA2, being consistent with the latency II type of EBV gene expression [36], [37], [38]. To test whether EBV-infected T and NK cells that proliferate in NOG mice retain this type of viral gene expression, we performed RT-PCR analysis in the spleen and the liver of mice that received PBMC from the CAEBV patient 3 (CD8 type). The results are shown in Figure 5A and demonstrate the expression of mRNAs coding for EBNA1, LMP1, LMP2A, and LMP2B, but not for EBNA2. Expression of the EBV-encoded small RNA 1 (EBER1) was also demonstrated. EBNA1 mRNAs transcribed from either the Cp promoter or the Wp promoter were not detected, whereas those transcribed from the Q promoter was abundantly detected. These results indicate that EBV-infected T cells retain the latency II pattern of latent EBV gene expression after engraftment in NOG mice. Similar analyses with NOG mice engrafted with EBV-infected NK cells also showed the latency II type of EBV gene expression (data not shown).

### NOG mice engrafted with EBV-infected T or NK cells produce high levels of human cytokines

In patients with CAEBV, high levels of cytokines have been detected in the peripheral blood and are thought to play important roles in the pathogenesis [20], [39], [40]. To test whether this hypercytokinemia is reproduced in NOG mice, we examined the levels of various human cytokines in the sera of transplanted mice using ELISA kits that can quantify human cytokines specifically. The results are shown in Figure 5B and indicate that the mice transplanted with PBMC of the patient 3 (CD8 type) or the patient 8 (NK type) contained high levels of RANTES, IFN-γ, and IL-8 in their sera.

### Engraftment of EBV-infected T and B cells derived from patients with EBV-HLH in NOG mice

**Figure 6:**
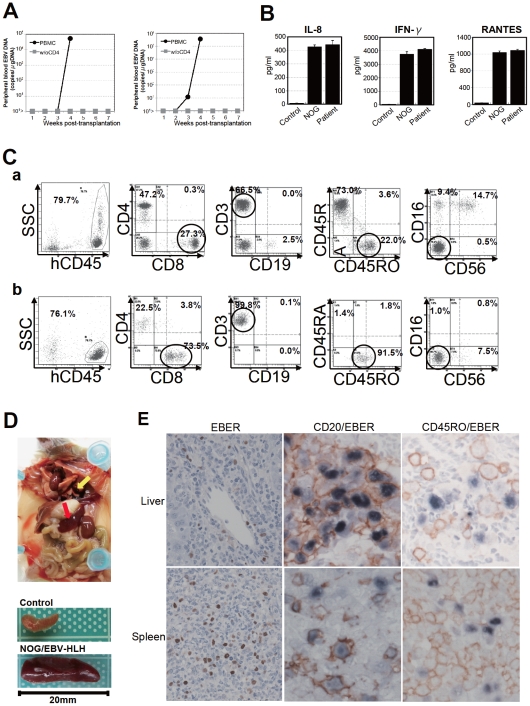
Engraftment of EBV-infected T and B cells in NOG mice transplanted with PBMC of patients with EBV-HLH. A. Peripheral blood EBV DNA load. Following transplantation with PBMC or PBMC devoid of CD4^+^ cells of the patient 11, EBV DNA was measured weekly by real-time PCR. Results of two mice prepared in an experiment are shown. B. Cytokine levels in the peripheral blood of the patient 12 and a mouse that received his PBMC. The levels of IL-8, IFN-γ, and RANTES were measured by ELISA in triplicates and the means and the standard errors are shown. A plasma sample of healthy person was used as a control. C. Immunophenotypic analyses on the peripheral blood lymphocytes of the EBV-HLH patient 10 (a) and a mouse that received his PBMC (b). Lymphocytes were gated by the pattern of the side scatter and the expression of human CD45, and analyzed for the expression of the indicated markers. The circles indicate the fractions that contained EBV DNA. D. Photograph of a mouse showing splenomegaly (red arrow) and hemorrhagic lesions (yellow arrow). Spleens excised from this mouse and a control mouse are shown at the bottom. E. Photomicrographs of the tissues of mice transplanted with EBV-HLH-derived PBMC. Liver and spleen tissues of a mouse transplanted with PBMC of the patient 11 were examined by EBER-ISH (left), double staining with an anti-human CD20 monoclonal antibody and EBER-ISH (middle), and double staining with an anti-human CD45RO monoclonal antibody and EBER-ISH (right). Original magnification ×600.

To extend the findings obtained from the CAEBV xenograft model to another disease with EBV^+^ T/NK lymphoproliferation, we transplanted NOG mice with PBMC isolated from patients with EBV-HLH. Characteristics of the four EBV-HLH patients examined in this study and the results of transplantation with their PBMC are summarized in Table 1. EBV DNA was detected in the peripheral blood three to four weeks post-transplantation and rapidly reached the levels of 1×10^4^ to 1×10^6^ copies/µg DNA (results of typical experiments are shown in Figure 6A). Similar to the findings in CAEBV, EBV DNA was not detected in the recipient mice, when CD4^+^ cell fraction was removed from PBMC (Figure 6A). Immunophenotypic analyses on the peripheral blood lymphocytes isolated from EBV-HLH patients and corresponding recipient mice revealed that cells of an identical immunophenotype (CD3^+^CD8^+^CD45RO^+^CD19^−^CD4^−^CD45RA^−^CD16^−^CD56^−^) were present and contained EBV DNA in both the patients and corresponding mice (Figure 6C and data not shown). The EBV DNA load observed in individual lymphocyte subsets in the patient 10 and a mouse that received his PBMC is shown as supporting data (Table S2). General condition of the recipient mice deteriorated consistently more quickly, with the loss of body weight (Figure S1), ruffling of hair, and general inactivity, than those mice engrafted with EBV-infected T or NK cells derived from CAEBV. The mice were sacrificed around four weeks post-transplantation for pathological analyses. Macroscopical observation revealed moderate to severe splenomegaly (Figure 6D) in the majority of recipient mice, and slight hepatomegaly in a limited fraction of them. A finding characteristic to these mice were massive hemorrhages in the abdominal and/or thoracic cavities, that were not seen in the mice transplanted with CAEBV-derived PBMC (Figure 6D and data not shown). These hemorrhagic lesions may reflect coagulation abnormalities characteristic to HLH. Histopathological analyses revealed a number of EBER^+^ cells in the spleen and the liver (Figure 6E) and quantification of EBV DNA in these tissues revealed 1.4×10^1^ to 2.4×10^2^ copies/µg of EBV DNA. When the tissues were examined by immunostaining and EBER ISH, the EBER^+^ cells were shown unexpectedly to be mostly CD45RO^-^ and CD20^+^ in all five transplantation experiments with four different patients, indicating that the majority of EBV-infected cells in these tissues are of the B-cell lineage (Figure 6E and data not shown). EBER^+^ large B cells were seen scattered among numerous reactive small T cells, most of which are CD8^+^, in the tissues of the spleen, liver, lungs and kidneys. A number of macrophages were also seen in these tissues. Fractionation of mononuclear cells obtained from the liver of a mouse transplanted with PBMC of the EBV-HLH patient 10, followed by real-time PCR, detected EBV DNA (1.4×10^1^ copies/µg DNA) only in the CD19^+^ B-cell fraction. In addition, an EBV-infected B lymphoblastoid cell line, but not an EBV-positive T cell line, could be established from this liver. Thus the presence of EBV in B cells were demonstrated by three independent methods in the tissues of EBV-HLH mice. Enzyme-linked immunosorbent assay revealed extremely high levels of human cytokines, including IL-8, IFN-γ, and RANTES, in the sera of both the original patients and the recipient mice (Figure 6B). The levels of IL-8 and IFN-γ were much higher than those observed in the peripheral blood of patients with CAEBV and mice that received their PBMC. Thus, NOG mice transplanted with EBV-HLH-derived PBMC are distinct from those transplanted with CAEBV-derived PBMC in the aggressive time course of the disease, internal hemorrhagic lesions, extremely high levels of IL-8 and IFN-γ in the peripheral blood, and the presence of EBV-infected B cells in lymphoid tissues.

## Discussion

The mouse xenograft models of CAEBV and EBV-HLH developed here represent the first recapitulation of EBV-associated T/NK lymphoproliferation in experimental animals. Previously, Hayashi and others inoculated rabbits with Herpesvirus papio and succeeded in the generation of T-cell lymphoproliferative disorder with pathological findings suggestive of EBV-HLH [41]. This model, however, is based on an EBV-related virus and not EBV itself, and therefore may contain features irrelevant to the original human disease. Although the CAEBV and EBV-HLH models described here exhibited some common features, including the abundant presence of EBV-infected T or NK cells in the peripheral blood, there were some critical differences between the two models, probably reflecting the divergence of the pathophysiology of the original diseases. First of all, in the EBV-HLH model mouse, EBV was detected mainly in B cells in the spleen and the liver, while it was found mainly in T cells in the peripheral blood. This makes an obvious contrast with the CAEBV model mouse, where EBV was detected in T or NK cells in both the peripheral blood and lymphoid tissues. We do not have an explanation for the apparent discrepancy in the host cell type of EBV infection between the peripheral blood and lymphoid tissues of the EBV-HLH model. It should be, however, noted that histopathology of EBV-HLH tissues has not been fully investigated and therefore it is still possible that significant number of EBV-infected B cells are present in the lymphoid tissues of EBV-HLH patients. Other differences between the two models include much higher plasma levels of IL-8 and IFN-γ more aggressive and fatal outcome, and internal hemorrhagic lesions in EBV-HLH model mice, probably reflecting the differences in the pathophysiology of the original diseases.

EBV-positive B-cell proliferation was not seen in CAEBV model mice even in long-term observation beyond twelve weeks. This seems puzzling since low but significant amount of EBV DNA was found also in B19^+^ B-cell fraction in most patients with CAEBV. It should be noted that EBV-infected T or NK cell lines could be established relatively easily from patients with CAEBV by adding recombinant IL-2 in the medium. In contrast, establishment of EBV-infected B LCLs from these patients has been extremely difficult. In fact, we could establish B-LCLs from a few patients with CAEBV only when their PBMC were cultured on feeder cells expressing CD40 ligand. Therefore, we speculate that in the particular context of CAEBV, both in the patient and the model mouse, proliferation of EBV-infected B cells are somehow inhibited by an unknown mechanism.

Analysis on the conditions of engraftment of EBV-infected T/NK cells using these new xenograft models revealed that EBV-infected T and NK cells of the CD8^+^ T, TCRγδT and CD56^+^ NK lineages and cell lines derived from them require CD4^+^ T cells for their engraftment in NOG mice. Only those EBV-infected cells and cell lines of the CD4^+^ T lineage could engraft in NOG mice on their own. These findings suggest that some factor(s) provided by CD4^+^ cells are essential for engraftment. Soluble factors produced by CD4^+^ T cells may be responsible for this function and we are currently examining cytokines, including IL-2, for their ability to support the engraftment of EBV-infected T and NK cells. It is also possible that cell to cell contact involving CD4^+^ cells is critical for engraftment. This dependence on CD4^+^ cells represents an interesting consistency with the previous finding that engraftment of EBV-transformed B lymphoblastoid cells in *scid* mice required the presence of CD4^+^ cells [42], [43]. It has been speculated that T cells activated by an EBV-induced superantigen may be involved in the engraftment of EBV-infected B lymphoblastoid cells in *scid* mice [44]. Although a similar superantigen-mediated mechanism might also be assumed in T- and NK-cell lymphoproliferation in NOG mice, the data of TCR repertoire analyses (Figure 1C and data not shown) show no indication for clonal expansion of Vβ13 T cells that are known to be specifically activated by the EBV-induced superantigen HERV-K18. It seems therefore unlikely that this superantigen is involved in the CD4^+^ T cell-dependent engraftment of EBV-infected T and NK cells. We expect CD4^+^ T cells and/or molecules produced by them may be an excellent target in novel therapeutic strategies for the treatment of CAEBV and EBV-HLH. In fact, administration of the OKT-4 antibody that depletes CD4^+^ cells in vivo efficiently prevented the engraftment of EBV-infected T cells. As a next step, we plan to test the effect of post-engraftment administration of OKT-4.

The dependence of EBV-infected T and NK cells on CD4^+^ T cells for their engraftment in NOG mice suggests the possibility that these cells are not capable of autonomous proliferation. Consistent with this notion, EBV-infected T and NK cell lines, including that of the CD4^+^ lineage, are dependent on IL-2 for their in vitro growth and do not engraft in either nude mice or *scid* mice when transplanted either s.c. or i.v (Shimizu, N., unpublished results). Clinically, CAEBV is a disease of chronic time course and patients carrying monoclonal EBV-infected T or NK cell population may live for many years without progression of the disease [15]. Overt malignant T or NK lymphoma usually develops only after a long course of the disease. Taking all these findings in consideration, we suppose that EBV-infected cells are not truly malignant at least in the early phase of the disease, even when they appear monoclonal. Because infection of EBV in T or NK cells is not unique to CAEBV and has been recognized also in infectious mononucleosis [45], [46], the critical deficiency in CAEBV may be its inability to immunologically remove EBV-infected T and NK cells. In this context, it should be emphasized that EBV-infected T or NK cells usually exhibit the latency II pattern of EBV gene expression and do not express EBNA3s, that possess immuno-dominant epitopes recognized by EBV-specific T cells [47]. EBV-infected T and NK cells are thus not likely to be removed by cytotoxic T cells as efficiently as EBV-infected B cells that express EBNA3s. The reported lack of cytotoxic T cells specific to LMP2A [17], one of the few immuno-dominant EBV proteins expressed in the virus-infected T and NK cells, may therefore seriously affect the host's capacity to control their proliferation. A genetic defect in the perforin gene was recently identified in a patient with clinical and pathological features resembling CAEBV, suggesting that defects in genes involved in immune responses can result in clinical conditions similar to CAEBV [48].

Engraftment of EBV-infected T and NK cells in NOG mice was in most cases accompanied by co-engraftment of un-infected cell populations. These un-infected cells might have been maintained and induced to proliferate by certain factors produced by EBV-infected T or NK cells. Abundant cytokines produced by these cells may be responsible for this activity. It is also possible that the proliferation of these un-infected cells represents immune responses. Experiments are underway to test whether these un-infected T cells contain EBV-specific cells. These un-infected T cells might also be reacting to host murine tissues. Intravenous injection of PBMC obtained from normal humans to immunodeficient mice including NOG mice has been shown to induce acute or chronic graft versus host disease (GVHD) [49], [50]. However, because much less PBMC were injected to mice in the present study as compared to those previous studies, it is not likely that major GVHD was induced in NOG mice transplanted with PBMC of patients with CAEBV or EBV-HLH.

CAEBV has been treated by a variety of regimens, including antiviral, cytocidal, and immunomodulating agents with more or less unsatisfactory results. Although hematopoietic stem cell transplantation, especially that with reduced intensity conditioning can give complete remission in a substantial number of patients [51], [52], it is still desirable to develop safer and more effective treatment, possibly with pharmaceutical agents. The xenograft model of CAEBV generated in this study may be an excellent animal model to test novel experimental therapies for the disease. In fact, the OKT-4 antibody that depletes CD4^+^ T cells in vivo gave a promising result implying its effectiveness as a therapeutic to CAEBV.

## Materials and Methods

### Ethics statement

Protocols of the experiments with materials obtained from patients with CAEBV and EBV-HLH and from control persons have been reviewed and approved by the Institutional Review Boards of the National Center for Child Health and Development and of the National Institute of Infectious diseases (NIID). Blood samples of the patients and control persons were collected after obtaining written informed consent. Protocols of the experiments with NOG mice are in accordance with the Guidelines for Animal Experimentation of the Japanese Association for Laboratory Animal Science and were approved by the Institutional Animal Care and Use Committee of NIID.

### Patients with CAEBV and EBV-HLH

Characteristics of the nine patients with CAEBV and the four patients with EBV-HLH examined in this study are summarized in Table 1. Diagnosis of CAEBV and EBV-HLH was made on the basis of the published guidelines [19], [53] and confirmed by identification of EBV-infected T or NK cells in their peripheral blood by flow cytometry and real-time PCR.

### NOD/Shi-*scid*/IL2Rγ^null^ (NOG) mice

Mice of the NOD/Shi-*scid*/IL-2Rγ^null^ (NOG) strain [22] were obtained from the Central Institute for Experimental Animals (Kawasaki, Japan) and maintained under specific pathogen free (SPF) conditions in the animal facility of NIID, as described [22].

### Transplantation of PBMC or their subfractions to NOG mice

PBMC were isolated by centrifugation on Lymphosepar I (Immuno-Biological Laboratories (IBL)) and injected intravenously to the tail vein of NOG mice at the age of 6–8 weeks. Depending on the recovery of PBMC, 1–4×10^6^ cells were injected to 2 to 4 mice in a typical experiment with a blood sample. For transplantation with individual cellular fractions containing EBV DNA, CD4^+^ T cells, CD8^+^ T cells, and CD56^+^ NK cells were separated with the IMag Cell Separation Systems (BD Pharmingen) following the protocol supplied by the manufacturer. To isolate γδT cells, CD19^+^, CD4^+^, CD8^+^, CD56^+^, and CD14^+^ cells were serially removed from PBMC by the IMag Cell Separation Systems. From the remaining CD19^−^CD4^−^CD8^−^CD56^−^CD14^−^ population, CD3^+^ cells were positively selected by the same kit and defined as the γδT cell fraction. To transplant PBMC lacking individual immunophenotypic subsets, CD19^+,^ CD4^+^, CD8^+^, CD56^+^ or CD14^+^ cells were removed from PBMC by the IMag Cell Separation Systems and the remaining cells were injected to mice. To prepare PBMC lacking γδT cells, CD19^+^, CD4^+^, CD8^+^, CD56^+^, and CD14^+^ cells isolated from PBMC in the process of obtaining γδT cell fraction (see above) were pooled and mixed with the CD19^−^CD4^−^CD8^−^CD56^−^CD14^−^ cells that did not react with anti-CD3 antibody. For complementation experiments, an EBV-containing cell fraction and the CD4^+^ cell fraction were isolated from a sample of PBMC as described above and the mixture of these two fractions were injected to NOG mice. The approximate numbers of injected cells are shown in Table 2.

### Analysis of immunophenotypes and TCR repertoire expression by flow cytometry

PBMC isolated from the patients and the recipient NOG mice as described above were incubated for 30 min on ice with a mixture of appropriate combinations of fluorescently labeled monoclonal antibodies. After washing, five-color flow-cytometric analysis was carried out with the Cytomics FC500 analyzer (Beckman Coulter). The following directly labeled antibodies were used: phycoerythrin (PE)-conjugated antibodies to CD3, CD8, and TCRα/β, fluorescein isothiocyanate (FITC)-conjugated antibodies to CD3, CD4, CD8, CD19, TCRVγ9, TCRVδ2, and TCRγ/δ, and Phycoerythrin Texas Red (ECD)-conjugated antibody to CD45RO from Beckman Coulter; PE-conjugated antibodies to CD16, CD40, and CD40L, and FITC-conjugated antibody to CD56 from BD Pharmingen. TCR Vβ repertoire analysis was performed with the Multi-analysis TCR Vβ antibodies Kit (Beckman Coulter) according to the procedure recommended by the manufacturer.

### Treatment of mice with the OKT-4 antibody

NOG mice were injected intravenously with 5×10^6^ PBMC isolated from the CAEBV patient 3 (CD8 type) or 8 (NK type) and were subsequently injected intravenously with 100 µg of the OKT-4 antibody on the same day of transplantation. Additional administration of the antibody was carried out by the same dose and route for the following three consecutive days. Peripheral blood EBV DNA load was then monitored every week. Mice were finally sacrificed four weeks post-transplantation and applied for pathological and virological analyses.

### Quantification of EBV DNA by real time PCR and analysis of EBV gene expression by RT-PCR

Quantification of EBV DNA was carried out by real-time quantitative PCR assay based on the TaqMan system (Applied Biosystems), as described [54]. Analysis of EBV gene expression by RT-PCR was carried out as previously described with the following primers [55]. EBNA1: sense, gatgagcgtttgggagagctgattctgca; antisense, tcctcgtccatggttatcac. EBNA2: sense, agaggaggtggtaagcggttc; antisense, tgacgggtttccaagactatcc. LMP1: sense, ctctccttctcctcctcttg; antisense, caggagggtgatcatcagta. LMP2A: sense, atgactcatctcaacacata; antisense, catgttaggcaaattgcaaa. LMP2B: sense, cagtgtaatctgcacaaaga; antisense, catgttaggcaaattgcaaa. EBER1: sense, agcacctacgctgccctaga; antisense, aaaacatgcggaccaccagc. Cp-EBNA1: sense,cactacaagacctacgcctctccattcatc; anti sense, ttcggtctcccctaggccctg. Wp/Cp-EBNA1: sense, tcagagcgccaggagtccacacaaat; antisense, ttcggtctcccctaggccctg. Qp-EBNA1: sense, aggcgcgggatagcgtgcgctaccgga; antisense, tcctcgtccatggttatcac. RT-PCR primers for β-actin were purchased from Takara (Osaka, Japan).

### Histopathology, EBER ISH, and immunohistochemistry

Tissue samples were fixed in 10% buffered formalin, embedded in paraffin, and stained with hematoxylin and eosin. For phenotypic analysis of engrafted lymphocytes, immunostaining for CD3, CD8 (Nichirei), CD45RO, and CD20 (DAKO) was performed on paraffin sections. EBV was detected by in situ hybridization (ISH) with EBV small RNA (EBER) probe. Immunohistochemistry and ISH were performed on an automated stainer (BENCHMARK XT, Ventana Medical Systems) according to the manufacturer's recommendations. To determine the cell lineage of EBV infected cells, paraffin sections were applied to double staining with EBER ISH and immunohistochemistry. Immediately following EBER ISH, immunostaining for CD45RO or CD20 was performed. Photomicrographs was acquired with a OLYMPUS BX51 microscope equipped with 40x/0.75 and 20x/0.50 Uplan Fl objective lens, a Pixera Penguin 600CL digital camera (Pixera), and Viewfinder 3.01 (Pixera) for white balance, contrast, and brightness correction.

### Quantification of cytokines

The levels of human IL-8, IFN-γ, and RANTES in plasma samples were measured with the Enzyme-linked immunosorbent assay (ELISA) kit provided by R&D Systems following instructions provided by the manufacturer.

### Accession numbers

The Swiss-Prot accession numbers for the proteins described in this article are as follows: P13501 for RANTES; P10145 for IL-8; P01579 for IFN-γ; P03211 for EBNA1; P12978 for EBNA2; P12977 for EBNA3; P03230 for LMP1; and Q66562 for LMP2. The DDBJ accession number for EBER is AJ315772.

## Supporting Information

Figure S1 Changes in the body weight of NOG mice transplanted with PBMC derived from patients with CAEBV or EBV-HLH. Body weight of the five CAEBV mice shown in Figure 1A (transplanted with PBMC from the patient 1, 3, 5, and 9, and with the CD4^+^ fraction from the patient 1, respectively) and two EBV-HLH mice shown in Figure 6A (both transplanted with PBMC from the patient 11) were recorded weekly.
(TIF)

Figure S2 Histopathological analysis of a control NOG mouse. A. a NOG mouse without xenograft. A 20-week old female NOG mouse was sacrificed and examined as a reference. No human cells are identified in these tissues. Upper panels: liver tissue was stained with hematoxylin-eosin (HE), antibodies specific to human CD3 or CD20, or by ISH with an EBER probe; the rightmost panel is a double staining with EBER and human CD45RO. Bottom panels: EBER ISH in the spleen, kidney, and small intestine. B. a NOG mouse transplanted with PBMC of a healthy EBV carrier. A six-week old female NOG mouse was transplanted with 5×10^6^ PBMC isolated from a normal EBV-seropositive person and sacrificed at eight weeks post-transplantation for histological analysis. Liver and Spleen tissues were stained with HE, antibodies specific to human CD3 or CD20, or by ISH with an EBER probe. No EBER-positive cells were identified in these tissues.Original magnification is ×200 for both A and B.
(TIF)

Table S1 EBV DNA load in lymphocyte subsets of a patient with CAEBV and a corresponding mouse derived from her PBMC.
(DOC)

Table S2 EBV DNA load in lymphocyte subsets of a patient with EBV-HLH and a corresponding mouse derived from his PBMC.
(DOC)
