# Supplementary figures and images for: Novel Mouse Xenograft Models Reveal a Critical Role of CD4+ T Cells in the Proliferation of EBV-Infected T and NK Cells
Source: PLoS Pathog. 2011 Oct 20;7(10):e1002326. doi: 10.1371/journal.ppat.1002326 (PMC3197618; doi:10.1371/journal.ppat.1002326)

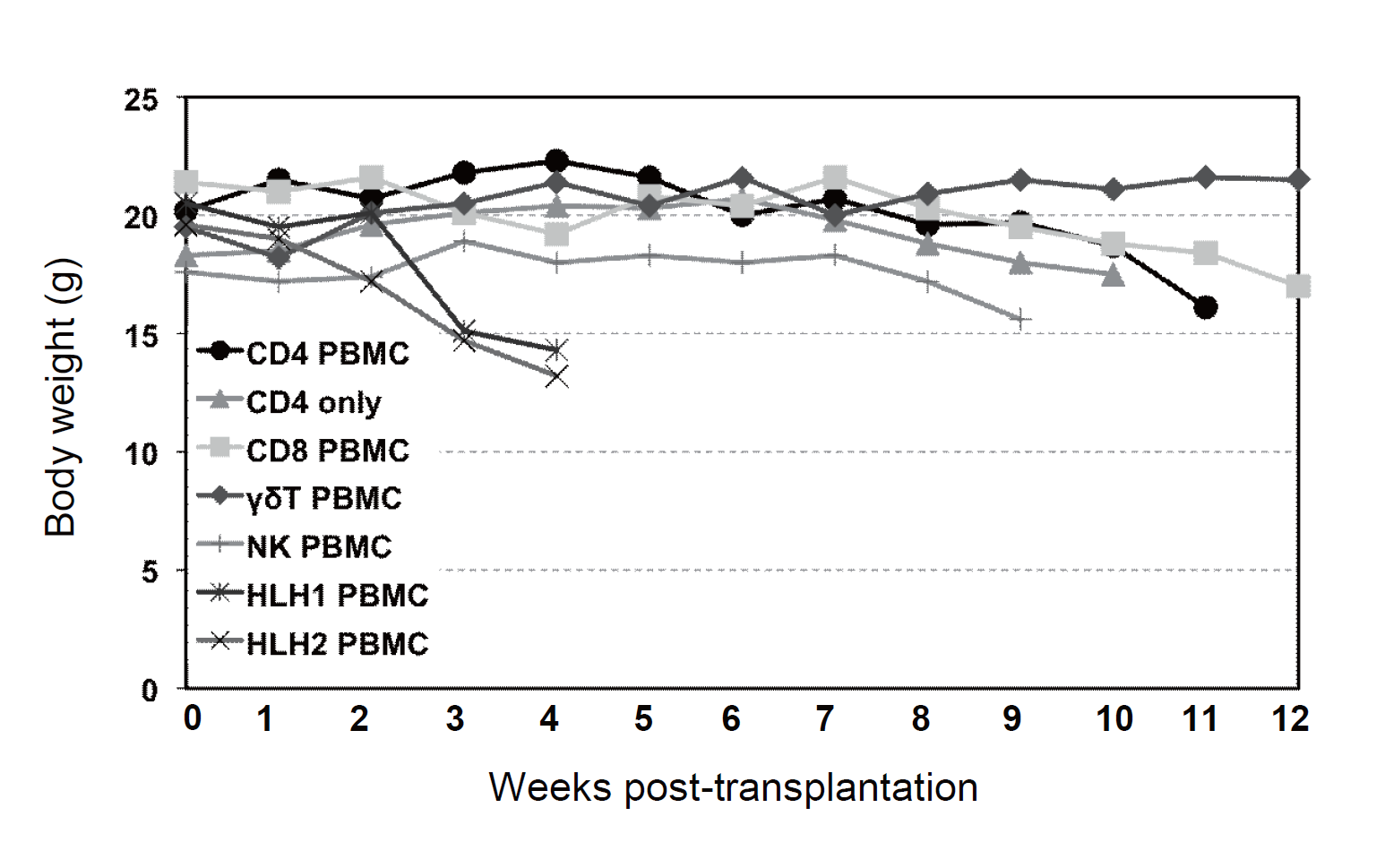

Supplement: Figure S1 — Changes in the body weight of NOG mice transplanted with PBMC derived from patients with CAEBV or EBV-HLH. Body weight of the five CAEBV mice shown in Figure 1A (transplanted with PBMC from the patient 1, 3, 5, and 9, and with the CD4+ fraction from the patient 1, respectively) and two EBV-HLH mice shown in Figure 6A (both transplanted with PBMC from the patient 11) were recorded weekly. (TIF) [file ppat.1002326.s001.tif]

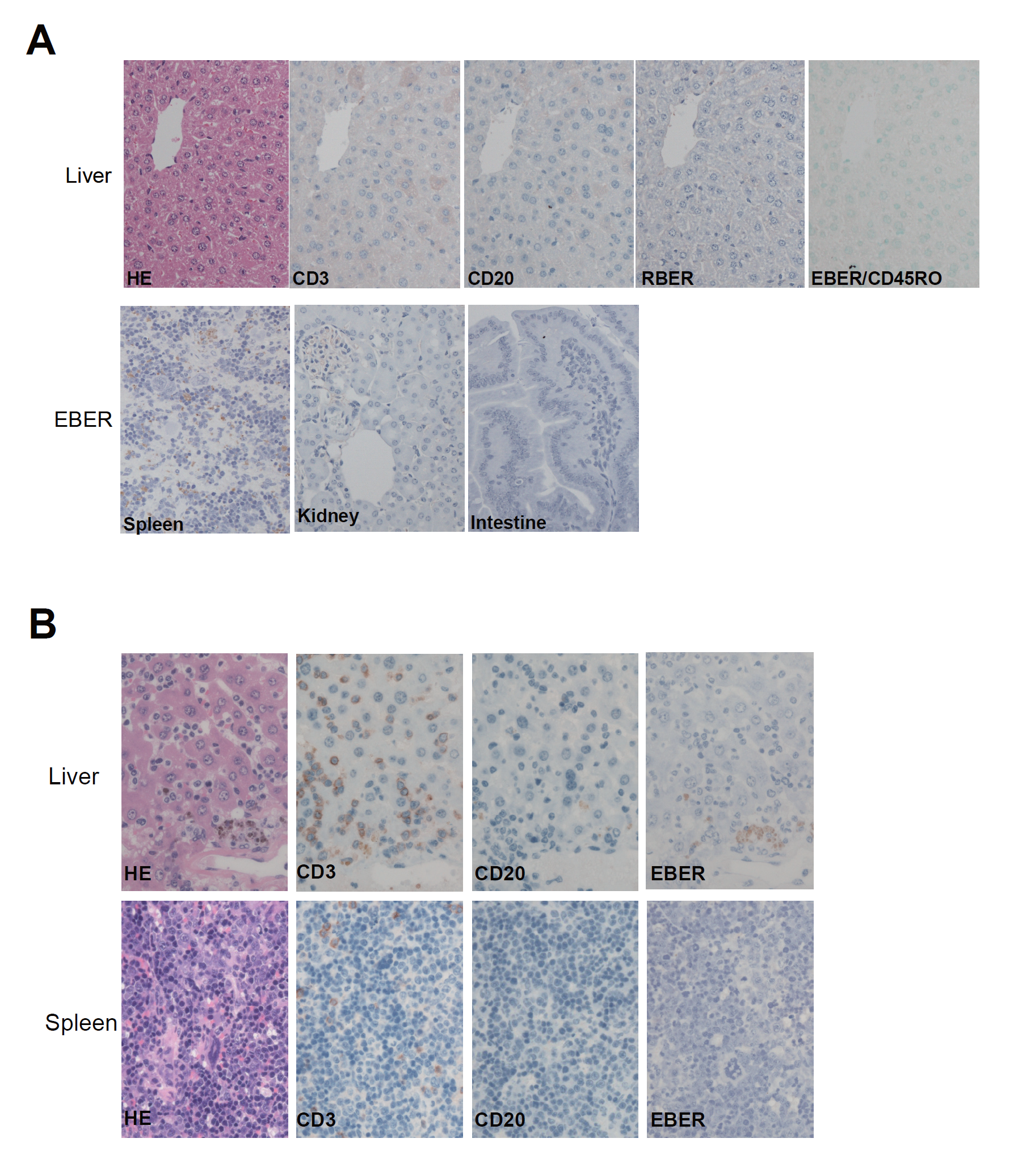

Supplement: Figure S2 — Histopathological analysis of a control NOG mouse. A. a NOG mouse without xenograft. A 20-week old female NOG mouse was sacrificed and examined as a reference. No human cells are identified in these tissues. Upper panels: liver tissue was stained with hematoxylin-eosin (HE), antibodies specific to human CD3 or CD20, or by ISH with an EBER probe; the rightmost panel is a double staining with EBER and human CD45RO. Bottom panels: EBER ISH in the spleen, kidney, and small intestine. B. a NOG mouse transplanted with PBMC of a healthy EBV carrier. A six-week old female NOG mouse was transplanted with 5×10^6^ PBMC isolated from a normal EBV-seropositive person and sacrificed at eight weeks post-transplantation for histological analysis. Liver and Spleen tissues were stained with HE, antibodies specific to human CD3 or CD20, or by ISH with an EBER probe. No EBER-positive cells were identified in these tissues.Original magnification is ×200 for both A and B. (TIF) [file ppat.1002326.s002.tif]
