# Supplementary material for: Novel Mouse Xenograft Models Reveal a Critical Role of CD4+ T Cells in the Proliferation of EBV-Infected T and NK Cells
Source: PLoS Pathog. 2011 Oct 20;7(10):e1002326. doi: 10.1371/journal.ppat.1002326 (PMC3197618; doi:10.1371/journal.ppat.1002326)
Supplement: Table S1 — EBV DNA load in lymphocyte subsets of a patient with CAEBV and a corresponding mouse derived from her PBMC. (DOC) [file ppat.1002326.s003.doc]

Table S1. EBV DNA load in lymphocyte subsets of a patient with CAEBV and

a corresponding mouse derived from her PBMC

| Lymphocyte subset | EBV DNA load in the patient^1^ | EBV ENA load in the mouse^1^ |
| --- | --- | --- |
| Whole PBMC | 2.1×10^5^ | 1.1×10^6^ |
| CD19^+^ | 4.2×10^2^ | UD.^2^ |
| CD4^+^ | UD. | UD. |
| CD8^+^ | 5.6×10^6^ | 2.5×10^6^ |
| CD56^+^ | UD. | UD. |
| CD14^+^ | UD. | UD. |
| Others^2^ | UD. | UD. |

^1^EBV DNA load in the peripheral blood is shown in copies/μg DNA.

^2^UD.: below detectable level (<10^1^ copies/μg DNA).

3Others: PBMC components that do not belong to the above subsets.
